# Editorial

**Published:** 1868-09

**Authors:** 


### Editorial.

Medical Colleges in this City.—The next Annual Lecture Term in Rush Medical College commences on the 30th of September. The introductory lecture will be given by Prof. E. Powell.

The next Annual Lecture Term in Chicago Medical College will commence on the 5th of October. The introductory lecture will be given by Prof. N. S. Davis. The laboratory for teaching analytical and practical chemistry in this College is now ready for the reception of students, and Prof. Wheeler informs us that a class is now entering upon its work in that department. The arrangements are very complete, and the instruction in practical chemistry will be as thorough as in the best schools in France and Germany.

Correction.—University of Nashville.—In a previous number of the Examiner, it was stated that "an effort seems to have been made to remove Professors Jennings, Eve, and Jones from their chairs in the Medical Department of the University. They denied the right of the rest of the Faculty to remove them, and appealed to the proper court. A decision has been recently rendered, sustaining their appeal, and enjoining the rest of the Faculty from interfering with them in the performance of their duties in the College." We made the statement from what purported to be the decision of the Judge or Chancellor having the case in charge, and, consequently, had no reason to doubt its correctness.

In the August number of the *Nashville Journal of Medicine*

*and Surgery* its correctness is denied, and the alleged facts in the case set forth. After stating the facts, however, the editor of that journal adds: "We know that Dr. Davis thought he had authority for his statement, but he knew it was one-sided, and a flat contradiction of its truth was before him which he saw proper to set aside, and he has thus constituted himself an agent for placing before the public a statement false in every particular."

Perhaps Dr. Bowling, in his sanctum in Nashville, may know better than we do what we have before us at any given moment of time here in Chicago; but we doubt it. Now, the truth is, we did make our statement on the *authority* of what purported to be the written decision of a Judge or Chancellor in Nashville. We did *not* know that any such decision was "one-sided." And we did not have before us, at that time, any contradiction of its truth, either *flat* or round. Such contradiction may have existed in a previous number of the *Nashville Journal*, or elsewhere; but if so, we had overlooked it. From the statements in the August number of the *Nashville Journal*, it appears that certain differences had arisen in the Faculty, by which it was claimed that Professors Jennings, Eve, and Jones had, by their own acts, vacated their chairs, and other men were appointed to fill them. The three named appealed to Chancellor Shackleford for an injunction, and it was granted, as we stated in the paragraph to which exceptions have been taken. But Judge Milligan, of the Supreme Court of Tennessee, issued a *supersedeas*, overruling the decision of the Chancellor. This latter part was unknown to us when we penned the former paragraph. And we are happy to end the matter by quoting the following, which is the last authoritative announcement in the *Nashville Journal:*—"Just as we are going to press, we are happy to announce that all suits against the new Faculty are withdrawn, and that there will be no further disturbance."

So may it be!

---

THE MEDICAL GAZETTE OF NEW YORK.—This sprightly little sheet had ceased to visit its readers until some of our exchanges had suggested its death. But its publication has been resumed, and the number for August 22d makes a better appearance than those preceding it.

---

HONORS.—The Royal Medical and Chirurgical Society of London recently conferred the title of Foreign Honorary Fellow on Dr. Samuel D. Gross, of Philadelphia.

---

Dr. WILLIAM MARSDEN, of Quebec, was recently made the recipient of a handsome present, from the citizens of that city, as a testimonial for his services in reference to the cholera.

---

BENNETT ECLECTIC MEDICAL COLLEGE.—We have received a circular announcing that a new college with the above title had been organized in this city. The ridiculous manner in which it prostitutes the name of Dr. Bennett, and the customary slanders upon the regular profession, show plainly the character of those engaged in the organization. *Eclectic* Colleges have been tried in Cincinnati and other cities, but with no very flattering degree of success.

---

NEBRASKA STATE MEDICAL SOCIETY.—The profession in Nebraska have recently organized a State Medical Society, with the following officers:—President, G. C. Monell, M.D.; Vice-President, N. C. Larsh, M.D.; Corresponding Secretary, G. C. Danise, M.D.; Permanent Secretary, S. D. Mercer, M.D.; Treasurer, D. Whittington, M.D. The next annual meeting is to be held in Nebraska City, on the first Tuesday in June, 1869.

---

MEDICAL COLLEGE CHANGES.—It is said that the Medical Department of Bowdoin College is to be removed to Portland, Maine.

William Warren Greene, M.D., late Professor of Surgery in the Michigan University, has taken up his residence in Portland, Maine.

---

Profs. Austin Flint, Senior and Junior, and Foster Swift, have resigned their places in the Long Island College Hospital.

New Sydenham Society's Publications.—Messrs. Lindsay & Blakiston, of Philadelphia, are the agents in this country for the sale of the publications of this Society. The subscription price for 1868 is ten dollars in currency.

---

Medical Department of University of Michigan.—The troubles in the Medical Department of this University are said to have been fully and satisfactorily settled.

---

Money Receipts to Aug. 28th.—Drs. Aug. Rhoads, $3: A. E. Van Deventer, 3; Wm. B. Hart, 3; John Sharp, 3; J: Guinan, 3; J. W. Duncan, 3; C. R. Parke, 3; P. S. Macdonald, 3; — Griffen, 1.50; L. T. Hewins, 5; R. S. Addison, 3.

---

### COOK COUNTY HOSPITAL, CITY OF CHICAGO.

### WINTER TERM OF CLINICAL INSTRUCTION, 1868–69.

The regular course of clinical lectures in this institution will commence on Friday, Oct. 2d, and continue for *nine months.*

The prosperity of this great public hospital, liberally supplied with the most adequate means for clinical and practical instruction, affords an extensive field for the study of every form of medical, surgical, and special disease.

During the year ending July 31st, 1868, over 1100 patients were treated in this hospital, and upwards of 100 important surgical operations were performed. In the Lying-in Department, there were 98 births.

The large number of autopsies—usually made in presence of the class—together with the pathological museum, gives an excellent opportunity for the study of morbid anatomy. The Dispensary, for out-patients, is organized to so classify diseases that every patient shall receive the most thorough attention. This, alone, furnishes a large number of interesting cases.

Lectures will be delivered as follows: Surgical Clinics, with operations, and Medical Clinics, with examinations at the bedside, on Tuesdays and Fridays; Eye and Ear Clinics, with operations, and Clinic on Diseases of Women and Children, on Saturdays. Commencing invariably at 1½ o'clock P.M., and continuing two hours.

It is the purpose of the medical staff to use and develop the immense resources of this institution, for the promotion of medical education; and to add whatever is possible to the means and facilities for medical study attainable in Chicago—the object for which they will labor being an exalted standard of professional knowledge among the students and such junior practitioners as may seek our city for this purpose.

Fee for admission to the Hospital, $5. Graduate practitioners free.

### LECTURES ON THE EYE AND EAR.

In addition to the course of lectures and operations on the Eye and Ear, embraced in the regular term of clinical instruction at the County Hospital, Chicago, Dr. Hildreth will also deliver a *Special Course* on these organs.

The theory of the ophthalmoscope; the analysis and treatment of "accommodation," astigmatism, and other optical and mechanical defects of the eye, will be fully dwelt upon. The methods of exploring the ear will be fully demonstrated.

The number of interesting cases in the Eye and Ear Wards of the hospital insures abundant opportunity for becoming familiar with ophthalmic and aural disease.

### THE CHARITABLE EYE AND EAR CLINIC,

A Free Dispensary, established by prominent citizens of Chicago, is likewise accessible to students.

The Hospital Ticket admits, without further charge, to all of the above.

---

Treatment of Diseases of the Heart.—Dr. S. O. Habershon, Physician to Guy's Hospital (Guy's Hospital Reports, 1867), lays down seven principles of treatment for diseases of the heart: 1st, to lessen its work; 2d, to insure regularity of action in avoiding all excitement; 3rd, to lessen the distention of the right heart by purgatives, diuretics, etc.; 4th, to prevent syncope attendant upon exhaustion; 5th, to strengthen the fibres of the heart by suitable out-door exercise; 6th, to prevent fibrillation of the blood by suitable remedies, for instance, carbonate of ammonia; and 7th, to prevent secondary complications, such as pneumonia, pleuritic effusion, etc.

### Mortality Report for the Month of July:—

### CAUSES OF DEATH.

| | |
|---|---|
| Abscess, | 1 |
| Accident, drowned | 16 |
| " by engine, | 1 |
| " fall, | 2 |
| " horse kick, | 1 |
| " poisoning, | 1 |
| " railroad, | 2 |
| " revolver, | 1 |
| " scalding, | 1 |
| Anæsarca, | 1 |
| " and diarrhœa | 1 |
| Apoplexy, | 10 |
| Apthia and diarrhœa, | 1 |
| Apoplexy, from over-exertion and heat, | 1 |
| Ascitis, abdominal, | 1 |
| Atelectasis pulmonium, | 1 |
| Births, premature, | 11 |
| " still, | 37 |
| " tedious, | 2 |
| Bowels, congestion of, and abortion, | 1 |
| " inflammation, | 8 |
| " perforation, | 1 |
| " uleeration of, | 1 |
| Brain, congestion of, | 6 |
| " disease of, | 2 |
| " inflammation | 9 |
| " tubercular disease | 1 |
| Catarrh, | 2 |
| Cholera infantum, | 273 |
| " convulsions, | 8 |
| " dysentery, | 1 |
| " foll. measles, | 1 |
| Cholerine, | 1 |
| Cholera infantum and phrenitis, | 1 |
| Childbirth, hemorrhage from, | 1 |
| Cholera morbus, | 7 |
| Childbirth | 1 |
| Convulsions, | 74 |
| " puerperal, | 2 |
| " sunstroke, | 1 |
| " urasmic, | 1 |
| " teething, | 15 |
| Croup, | 3 |
| Cyanosis, | 1 |
| Debility, | 8 |
| " and old age, | 1 |
| Delirium tremens, | 6 |
| Diphtheria, | 2 |
| Diabetes milletus, | 1 |
| Diarrhœa, | 34 |
| " chronic, | 6 |
| " & convulsions | 5 |
| Dropsy, | 2 |
| Dysentery, | 17 |
| " chronic, | 1 |
| " complicated with pneumonia, | 1 |
| " & teething, | 1 |
| Endocarditis, | 1 |
| Enteritis, | 4 |
| " complicated with meningitis, | 2 |
| Enteritis, complicated cholera infantum, | 1 |
| Encephatitis, | 5 |
| Enterocolites | 3 |
| Epistaxis, | 1 |
| Epilepsy | 2 |
| " superinduced by heat, | 1 |
| Erysipelas, | 1 |
| " foll. measles, | 1 |
| " traumatic, | 1 |
| Exhaustion from exposure, | 2 |
| Fever, congestive, | 2 |
| " " and diarrhœa, | 1 |
| " puerperal, | 4 |
| " scarlet, | 11 |
| " " and diptheria, | 1 |
| " typhoid, | 9 |
| " and gastromalaxy, | 1 |
| Gastritis | 1 |
| " and intemperance, | 1 |
| Gastroenteritis, | 1 |
| Hepatitis, | 1 |
| Heart, disease of | 2 |
| " dropsy of | 1 |
| " organic disease | 2 |
| " hypertrophy of | 1 |
| " valvular disease | 2 |
| Hemorrhage, internal, | 1 |
| Hernia, strangulated | 2 |
| Hydrocephalus | 10 |
| " acute | 7 |
| Intemperance and exposure, | 2 |
| Idigestion, | 1 |
| Inanition, | 9 |
| Ileus, | 1 |
| Intestines, inflammation of | 1 |
| " injuries of | 1 |
| Jaundice, | 1 |
| Kidneys, Bright's disease of | 1 |
| Leg, tumor of | 1 |
| Lightning, struck by | 3 |
| Liver and kidneys fatty degeneration of | 1 |
| Liver, cirrhosis of | 1 |
| " inflammation of | 1 |
| Lungs, congestion of | 1 |
| " " of at childbirth, | 1 |
| " congestion of and measles, | 1 |
| Meningitis | 15 |
| " acute, | 1 |
| " foll. diarrhœa | |
| " cerebro spinal, | 8 |
| " tubercular, | 4 |
| Mania, puerperal | 1 |
| Manslaughter, | 1 |
| Measles, | 7 |
| Metrophibitis, puerperal, | 1 |
| Miliaria puperanum, retroception of | 1 |
| Mouth, canker sore of | 1 |
| Myetitis, acute | 1 |
| Nutrition, defective | 1 |
| Œdema glottde, | 1 |
| Old age, | 4 |
| " and debility, | 3 |
| " " paralysis, | 1 |
| Palate, fissure of, | 1 |
| Paralysis, | 3 |
| Parotitis, | 1 |
| Paralysis and old age, | 1 |
| Pericarditis | 1 |
| Periostitis of inferior maxillary, | 1 |
| Pistolshot through abdomen, | 1 |
| Pleuritis, double, and emboli in heart, | 1 |
| Poisoned wound from snake bite, | 1 |
| Phthisis, acute, | 1 |
| " pulmonalis, | 32 |
| " " & abortion, | 1 |
| Pyæmia, | 1 |

| | | | | | |
|---|---|---|---|---|---|
| Pneumonia, | 17 | Sunstroke, | 19 | Uræmia, | 2 |
| " complicated | | Small-pox, | 2 | Uterus, cancer of, | 1 |
| with measles, | 3 | Stomach, congestion of | 1 | " hemorrhage of, | 1 |
| " typhoid, | 3 | Stomatitis, | 2 | Whooping cough, | 6 |
| " and inflamma- | | Tabes mesenterica, | 19 | " " & con- | |
| tion of bowels, | 1 | Tetanous traumaticus, | 1 | vulsions, | 3 |
| Rheumatism, inflamma- | | Teething, | 27 | Whooping cough and | |
| tory, | 1 | " and diarrhœa, | 5 | diarrhœa, | 4 |
| Scrofula, | 2 | " and meningitis, | 1 | Unknown, | 1 |
| Septæmia, | 1 | Tumor, abdominal, | 1 | | |
| Suicide, | 4 | " ovarian, | 3 | Total | 896 |

COMPARISON.

Deaths in July, 1868, 896 | Deaths in July, 1867, 597 | Increase, 299

Deaths in June, 1868, 305 | Increase, 591

AGES.

| | | | | | |
|---|---|---|---|---|---|
| Under 5 | 654 | 40 to 50 | 40 | 90 to 100 | 0 |
| 5 to 10 | 27 | 50 to 60 | 24 | 100 to 110 | 0 |
| 10 to 20 | 24 | 60 to 70 | 15 | Unknown | 2 |
| 20 to 30 | 45 | 70 to 80 | 11 | | |
| 30 to 40 | 54 | 80 to 90 | 0 | Total | 896 |

| | | |
|---|---|---|
| Males, 510 | Females, 385 | Total, 896 |
| Single, 765 | Married, 131 | Total, 896 |
| White, 891 | Colored, 5 | Total, 896 |

NATIVITY.

| | | | | | |
|---|---|---|---|---|---|
| Chicago | 555 | England | 13 | Scotland | 5 |
| Other parts U. S. | 92 | Germany | 110 | Sweden | 26 |
| Austria, | 2 | Holland | 2 | Switzerland | 1 |
| Bohemia | 5 | Ireland | 49 | Unknown | 5 |
| Canada | 8 | Norway | 16 | | |
| Denmark | 1 | Poland | 4 | Total | 896 |
| France, | 1 | Atlantic Ocean, | 1 | | |

MORTALITY BY WARDS FOR THE MONTH.

| Ward. | Mortality. | Pop. in 1868. | One death in | | Ward. | Mortality. | Pop. in 1868. | One death in | |
|---|---|---|---|---|---|---|---|---|---|
| 1 | 10 | 11,991 | 1,199 | 1-10 | 14 | 63 | 14,168 | 224 | 6-7 |
| 2 | 19 | 13,739 | 723 | 1-9 | 15 | 65 | 20,429 | 314 | 2-7 |
| 3 | 43 | 16,620 | 386 | 1-2 | 16 | 31 | 16,011 | 519 | 2-3 |
| 4 | 42 | 16,499 | 392 | 4-5 | Bridewell, | 2 | | | |
| 5 | 36 | 13,434 | 373 | 1-6 | County hosp. | 19 | | | |
| 6 | 50 | 12,507 | 250 | 1-7 | Chi. River, | 3 | | | |
| 7 | 88 | 21,957 | 249 | 1-9 | Mercy Hosp. | 2 | | | |
| 8 | 59 | 14,003 | 237 | 1-3 | Yard of R. I. Railroad, | 1 | | | |
| 9 | 57 | 18,050 | 234 | 1-5 | | | | | |
| 10 | 28 | 13,644 | 487 | 2-5 | Soldier's hom. | 1 | St. Joseph's Orph. As. | | 6 |
| 11 | 50 | 13,317 | 266 | 1-3 | Marine hos. | 1 | Home for Friendless, | | 2 |
| 12 | 105 | 14,739 | 141 | 1-3 | Orphan asyl. | 1 | Immigrants, | | 56 |
| 13 | 49 | 11,113 | 226 | 4-5 | Lake Michi. | 7 | Police Stat. 12 St., | | 1 |

Total, 896

DRUGGISTS RENEWING PRESCRIPTIONS.—The Academy of Medicine, New York, after a long and impartial discussion of the various points at issue, adopted unanimously the following preamble and resolution, and referred them to the State Medical Society:

WHEREAS, The attention of this Academy has been called to the repetition of prescriptions, containing active ingredients, by druggists, without the written order of physicians; and whereas, serious consequences to patients are liable to ensue; therefore,

*Resolved*, That we respectfully request the druggists of this city not to repeat such a prescription without the written order of the physician, he being the only competent judge of the propriety or necessity of such renewal.

---

UTERINE INJECTIONS.—At a recent meeting of the Imperial Academy of Medicine, the subject of uterine injections, which of late has occupied the attention of that learned body, was again briefly discussed, and MM. Gosselin, Depaul, and Ricord condemned this method of therapeutics, which, despite the perfection of the instruments adopted, may determine an attack of metro-peritonitis, which, according to the declaration of M. Gosselin, has already occurred in the service of M. Jobert.—*L'Evenement Medicale.*

---

MUSICAL BULLET PROBE.—At the Paris Exposition there was exhibited a probe for announcing audibly the presence of a bullet in a wound. If the points of the instrument came in contact with a metallic body, an electrical circuit was made and a small bell rang.

An ozone generator was also exhibited, made of flat plates of glass coated with tin foil and electrified by a Ruhmkoff coil. A stream of air driven through the apparatus came out so highly charged with ozone as to be irrespirable, and possessing the power of bleaching paper, linen, soiled engravings, and so forth. This discovery is spoken of as likely to be of much practical value in the arts.—*Pacific Medical and Surgical Journal.*

---

GOSSYPIUM AS AN EMMENAGOGUE AND PARTURIFACIENT.—Dr. Bellamy, of Columbus, Ga., says of the common cotton-plant, gossypium (*Atlanta Med. and Surg. Journal*):—"I am fully satisfied, from the experiments and impartial tests I have given the remedy, that it is fully equal, if not superior, to ergot in promoting the various functions of the uterine organs. I look upon it as a sure, speedy, and safe remedy, not only for difficult, painful, contracted labors, but also to control all the irregularities of females, and to alleviate their peculiar monthly sufferings. It is very certain that its effects are so powerful upon the uterine system as to produce miscarriage, if administered during pregnancy. I feel that its merits cannot be too highly extolled, and deem it too valuable a remedy to remain hidden in the depths of obscurity. I consider it preferable to ergot. The proper time to gather the root is when it is as old as possible, without being injured by the severe frosts: therefore, it is best when gathered during the months of October and November."

---

Deaths from Sunstroke.—An examination of the statistics of death from sunstroke in the city of New York, gives the following exhibit: In August, 1853, 224 persons died from sunstroke; in 1863, there were 135 deaths from sunstroke, and during the present year up to Saturday, the 18th ult., there were no less than 883 deaths from heat alone, as reported by the papers.—*Ex.*

---

An Alleged Preservative against the Cattle Plague.—Chloride of copper is now extensively used in Germany against the cattle plague, or rather as a preservative. The *modus eperandi* is as follows:—

Take green crystallized chloride of copper, eight gram., spirits of wine, two kilog., and dissolve. With this solution impregnate a pad of cotton, lay it on a plate, and set fire to it in the centre of the stable, turning the animals' heads towards the flame, so as to make them breathe the fumes. This operation is performed morning and evening, burning one pad for every three heads of cattle. At night, a spirit-lamp filled with this solution is lighted in the stable. To prevent accidents, the flame is surrounded with wire gauze. The liquid is also administered internally, with the addition of fifteen gram. of chloroform for the above quantity. A teaspoonful of this is put into the animals' drink three times a day. As a further precaution, the litters are watered with the same solution.

---

Effect of Digitalis on the Mind.—It is a popular opinion that digitalis acts injuriously upon the *memory*, and that it hastens childishness in elderly persons. Undoubtedly it has a peculiar and specific influence on the mind, probably through its remarkable power over the circulatory system. It has recently been used in Germany with success in the treatment of hallucinations. In the *Allg. Ztschr. fur Psychiatrie* a case of a woman is reported, aged 38 years, who was constantly troubled with the appearance of devils and imps, brandishing flames and threatening to strangle her. She was placed on digitalis (℈ss., aquæ ℥v., ℨj. t. d.), and in two or three months was quite restored.

The real effects of the drug on mental action should be carefully studied.

